# Multiple Active Contours Guided by Differential Evolution for Medical Image Segmentation

**DOI:** 10.1155/2013/190304

**Published:** 2013-07-25

**Authors:** I. Cruz-Aceves, J. G. Avina-Cervantes, J. M. Lopez-Hernandez, H. Rostro-Gonzalez, C. H. Garcia-Capulin, M. Torres-Cisneros, R. Guzman-Cabrera

**Affiliations:** Universidad de Guanajuato, Division de Ingenierias Campus, Irapuato-Salamanca, Carretera Salamanca-Valle de Santiago, Km 3.5+1.8 Km Comunidad de Palo Blanco, C.P. 36885, Salamanca, GTO, Mexico

## Abstract

This paper presents a new image segmentation method based on multiple active contours guided by differential evolution, called MACDE. The segmentation method uses differential evolution over a polar coordinate system to increase the exploration and exploitation capabilities regarding the classical active contour model. To evaluate the performance of the proposed method, a set of synthetic images with complex objects, Gaussian noise, and deep concavities is introduced. Subsequently, MACDE is applied on datasets of sequential computed tomography and magnetic resonance images which contain the human heart and the human left ventricle, respectively. Finally, to obtain a quantitative and qualitative evaluation of the medical image segmentations compared to regions outlined by experts, a set of distance and similarity metrics has been adopted. According to the experimental results, MACDE outperforms the classical active contour model and the interactive Tseng method in terms of efficiency and robustness for obtaining the optimal control points and attains a high accuracy segmentation.

## 1. Introduction

Computed tomography (CT) scanning and magnetic resonance imaging (MRI) are widely used in medical tests since they represent a noninvasive and painless modalities for the diagnosis of cardiac disease. In clinical practice, the process performed by a cardiologist on medical images can be subjective, labor intensive, and susceptible to errors because it is based on a visual examination followed by a manual delineation of the human organ. Consequently, the application of computational techniques in order to obtain a more efficient and accurate image segmentation within an acceptable time plays an essential role.

In medical image analysis, the automatic segmentation of human organs is an important and challenging task. In the literature, several techniques have been reported for this purpose such as, region growing in pelvic injuries [1], improved watershed transform for tumors in mammograms [2], enhanced suppressed fuzzy c-means to work with brain magnetic resonance images [3], wavelet transform in dermoscopic images [4], templates for atlas in radiotherapy [5], and active contour models (ACMs) in mammographic images [6, 7]. This method was introduced by [8] and it is an energy-minimizing spline that consists of control points also called snaxels. This spline evolves through the evaluation of internal and external forces according to the shape of the object to be segmented. ACM has been extensively used in medical applications such as segmentation of human prostate [9], intravascular ultrasound images [10], breast lesions [11], and breast tumors [12].

In the traditional implementation of active contour model there exist two main weaknesses. The first drawback is the initialization of control points, which must be close to the object of interest to achieve a favorable segmentation otherwise failure of convergence will occur. The second drawback is the propensity to stagnate in local minima giving an inaccurate convergence to the boundaries of the object. To solve these disadvantages some improvements have been suggested to adapt different methods to work together with ACM including statistical methods [13, 14], graph cut [15], population based-methods such as particle swarm optimization (PSO) working with polar sections [16], static large searching windows [17] and by adapting the PSO velocity equation [18], genetic algorithms [19, 20], and differential evolution [21]. The performance of the population based-methods working together with ACM is very suitable according to the tests since the active contour becomes more stable, robust, and efficient in local minima problem.

Differential evolution (DE) is a stochastic and population-based optimization method similar to evolutionary algorithms suggested by [22, 23]. DE has become very popular for solving global optimization problems with nondifferentiable and nonlinear functions with a fast convergence. The efficiency and robustness of the DE method directly depend on the settings of the control parameters such as population size, selection method, differentiation factor, and the crossover probability constant which controls the number of generated solutions for each individual through generations. As DE is easy to implement, not computationally expensive and it is highly efficient solving optimization problems, it has been used in many real-world applications such as text summarization [24], design of reconfigurable antenna arrays [25], job shop scheduling problem [26], blade design of wind turbines [27], and in the parameter estimation for a human immunodeficiency virus (HIV) [28].

In this paper, we introduce a novel image segmentation method based on multiple active contours guided by differential evolution optimization technique, which divides the object of interest in polar sections. Each polar section has a population of individuals represented by control points to perform its particular search strategy in order to find the optimal control point (snaxel). Since the proposed method can appropriately overcome the drawback of initialization of the traditional ACM and the inaccurate convergence on the concave boundaries of an object, MACDE also addresses the problem of segmenting the human heart and the human left ventricle from datasets of sequential CT and magnetic resonance images, respectively. Finally, to visualize the segmentation results of CT images a 3D reconstruction approach of the human heart is presented.

The structure of this work is as follows. In Section 2, the fundamentals of active contour model and differential evolution are presented. In Section 3, the proposed MACDE method is introduced, along with a set of validation metrics to evaluate its performance. The experimental results are discussed in Section 4, and from the similarity metrics, conclusions are presented in Section 5.

## 2. Background

In this section, the fundamentals of the active contour model and differential evolution optimization technique are explained in detail. 

### 2.1. Active Contour Model

Active contour model (ACM), also known as snake, is a parametric curve, which can move within the spatial domain of an image where it was assigned. The snake is defined by *p*(*s*, *t*) = (*x*(*s*, *t*), *y*(*s*, *t*)),  *s* ∈ [0,1], where *t* represents the time parameter whereby the curve evolves in order to minimize the total energy function given by

(1) $$\begin{array}{c} E_{\text{snake}}=\int_{0}^{1}\left[E_{\mathrm{int}}\left(p\left(s,t\right)\right)+E_{\text{ext}}\left(p\left(s,t\right)\right)\right]\text{d}s. \end{array}$$

The above-mentioned energy function consists of two components: *E*_int⁡_ that represents the internal energy and *E*_ext_ the external energy. The internal energy presented in (2) is composed by the first derivative of *p*(*s*) guided by the curve tension parameter *α*(*s*) and the second derivate of *p*(*s*) controlled by the rigidity parameter *β*(*s*). This energy keeps the search performed by the control points within the spatial image domain and also it controls the shape modification of the parametric curve as follows:

(2) $$\begin{array}{c} E_{\mathrm{int}}\left(p\left(s,t\right)\right)=\frac{1}{2}\left[\alpha \left(s\right){\left|\frac{\partial p\left(s\right)}{\partial s}\right|}^{2}+\beta \left(s\right){\left|\frac{\partial^{2}p\left(s\right)}{\partial s^{2}}\right|}^{2}\right]. \end{array}$$

The external energy represented by (3) is given by the particular features of the search space, where *γ* is a weight parameter and ∇*I*(*p*(*s*)) is the surface gradient computed at *p*(*s*) achieving the optimal solution by solving the Euler equation (4), when both external and internal energies become stable

(3) $$\begin{array}{c} E_{\text{ext}}\left(p\left(s\right)\right)=-\gamma {\left|\nabla I\left(p\left(s\right)\right)\right|}^{2}, \end{array}$$

(4) $$\begin{array}{c} \nabla E_{\text{ext}}-\alpha \frac{\partial^{2}p\left(s\right)}{\partial s^{2}}+\beta \frac{\partial^{4}p\left(s\right)}{\partial s^{4}}=0. \end{array}$$

In the discrete computational implementation of ACM, the snake is composed by a number *n* of discrete points {*p*_*i*_ | *i* = 1,2,…, *n*}. The discrete formulation of internal and external energies are approximated by (5) and (6), respectively, where *q*_*i*,*j*_ represents the current snake control point *p*_*i*_ and *j* the index point within its searching static window. Accordingly, the local energy function is given by (7), in which the minimization process is iteratively performed by using (8), where *W*_*i*_ is the predefined searching window for the control point *p*_*i*_ and *k*_*i*_ is obtained by minimizing the local energy function [17]:

(5) $$\begin{array}{c} E_{\mathrm{int}}=\frac{1}{2}\left[\alpha \left(s\right){\left|q_{i,j}-p_{i-1}\right|}_{2}^{2}+\beta \left(s\right){\left|p_{i-1}-2q_{i,j}+p_{i+1}\right|}_{2}^{2}\right], \end{array}$$

(6) $$\begin{array}{c} E_{\text{ext}}=-\gamma {\left|\nabla I\left(q_{i,j}\right)\right|}_{2}^{2}, \end{array}$$

(7) $$\begin{array}{c} E_{i,j}=E_{\mathrm{int}}+E_{\text{ext}}, \end{array}$$

(8) $$\begin{array}{c} E_{\text{snake}}=\sum_{i=1}^{n}E_{i,k_{i}},\;k_{i}=\arg\;\underset{j}{\min}\left(E_{i,j}\right),\;\;j\in W_{i}. \end{array}$$

There exist two main weaknesses in the traditional implementation of ACM. Firstly, sensitivity to the initial positioning of the control points (snaxels) and secondly, the propensity to stagnate in local minima deflecting the snake of the optimum edge of the object of interest. In order to overcome the aforementioned drawbacks of the ACM, a population-based technique such as differential evolution optimization (DE) has been adopted, which is described in the following Section 2.2.

### 2.2. Differential Evolution

Differential evolution (DE) is a stochastic real-parameter heuristic proposed by [22, 23] for numerical global optimization problems similar to standard evolutionary algorithms. DE starts with a set of randomly initialized potential solutions, called individuals *X* = {*x*_1_, *x*_2_,…, *x*_*Np*_}, where *Np* is the population size. These individuals are gradually improved by applying different variation operators and the solution is chosen to be the individual with the best fitness according to an objective function.

The fundamental idea behind DE algorithm consists of three evolutionary principles: mutation, crossover, and selection on the floating-point encoding. The mutation step creates a mutant vector *V*_*i*,*g*+1_ at each generation *g* based on the distribution of the current population {*X*_*i*,*g*_ | *i* = 1,2,…, *Np*} by performing the classical mutation strategy presented in

(9) $$\begin{array}{c} V_{i,g+1}=X_{r1,g}+F\left(X_{r2,g}-X_{r3,g}\right),\;r1\neq r2\neq r3\neq i, \end{array}$$

where *r*1, *r*2, and *r*3 represent the indexes of three individuals mutually different and uniformly selected from the set {1,…, *Np*} and the *F* represents the differentiation factor also known as scaling or mutation factor parameter. After the mutation process, the crossover operator is applied based on (10), to create the trial vector *U*_*i*,*g*+1_

(10) $$\begin{array}{c} U_{i,g+1}=\{\begin{array}{cc} V_{i,g+1}, & \text{if}\;\;r\leq \text{CR},\\ X_{i,g}, & \text{if}\;\;r>\text{CR}, \end{array} \end{array}$$

where *r* is a uniform random value on the interval (0,1), which is compared with the CR (crossover rate) parameter. If *r* is bigger than CR, the current information of individual *X*_*i*,*g*_ is conserved, otherwise the values from the mutant vector *V*_*i*,*g*+1_ are copied to the trial vector *U*_*i*,*g*+1_. Subsequently, the selection procedure is applied by using (11) to minimization process. This procedure selects, according to a fitness function, the better one between the trial vector *U*_*i*,*g*+1_ and the individual *X*_*i*,*g*_. The selected vector is used to replace the current individual in the next generation:

(11) $$\begin{array}{c} X_{i,g+1}=\{\begin{array}{cc} U_{i,g+1}, & \text{if}\;\;f\left(U_{i,g+1}\right)<f\left(X_{i,g}\right),\\ X_{i,g}, & \text{otherwise}. \end{array} \end{array}$$

According to the previous description, the classical DE algorithm is described by using the following procedure.

1. Initialize number of generations *G*, population size *Np*, value of differentiation factor *F*, and value of crossover rate CR.
2. Initialize each individual *X*_*i*_ by generating random candidate solutions.
3. For each individual *X*_*i*,*g*_, where *g* = {1,…, *G*}:
  - compute *V*_*i*,*g*+1_ by using the mutation step (9);
  - assign *U*_*i*,*g*+1_ according to the crossover operator (10);
  - update *X*_*i*,*g*+1_, if *U*_*i*,*g*+1_ is better than *X*_*i*,*g*_ by applying the selection step (11).
4. If stopping criterion is satisfied (e.g., stability or number of generations), then stop.

## 3. Proposed Image Segmentation Method

The proposed MACDE method based on differential evolution and multiple active contours is described in Section 3.1. In addition, to obtain a quantitative evaluation of the segmentation results obtained from the proposed method, the set of similarity metrics is explained in Section 3.2.

### 3.1. Multiple Active Contours Guided by Differential Evolution (MACDE)

Because of the classical ACM weaknesses discussed above, differential evolution is adopted to solve the local minima drawback by guiding the convergence of multiple active contours on a polar coordinate system similar to [16]. Since DE is directly applied in the segmentation task performed by MACDE, the advantages of robustness, low computational time, and efficiency are preserved. The proposed method presents three main advantages on the initialization process, which must be considered to adapt it to the shape of the object of interest. Firstly, the initial contours can be automatically defined in a circular or elliptical shape. Secondly, the number of snaxels (individuals) can be modified according to the number of polar sections in which the object of interest is divided. The third advantage is the origin or seed point created interactively by the user to generate all the snaxels automatically on the constrained spatial domain of the object of interest. This latter advantage allows to use the proposed method in the segmentation of stacks of sequential CT and MR images in order to obtain a 3D reconstruction approach of human organs by just reproducing the origin point through the set of images along with the predefined parameters.

**Figure 1:**
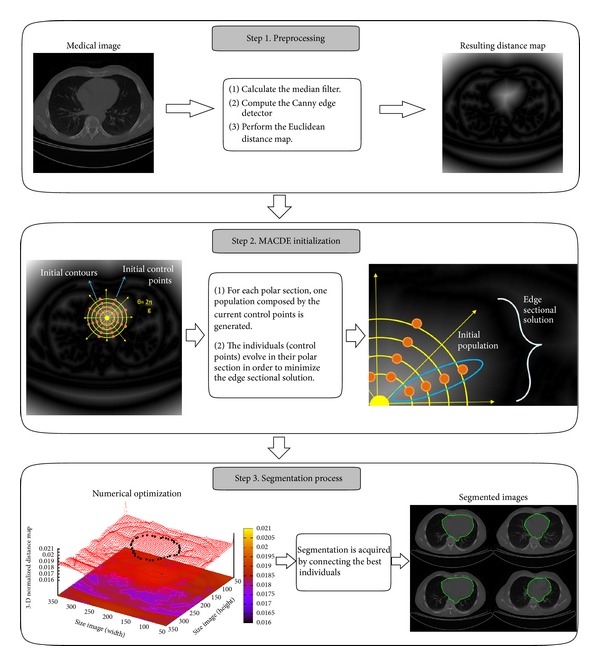
Process of the proposed MACDE image segmentation method.

The procedure of MACDE segmentation method consists of three steps and it is illustrated in Figure 1. The preprocessing stage reduces noise from the image by using a 2D median filter (3 × 3 window size), followed by the Canny edge detector (*σ* = 1.3, *T*_*l*_ = 10.0, and *T*_*h*_ = 30.0) to detect the boundary between the background and regions of interest. These parameters have been experimentally tuned to preserve the real edges in the image, since these can affect the segmentation result. The final step in this stage is to compute the Euclidean distance map (EDM) according to [29]. The EDM is used to perform the minimization process because it represents a potential surface, where high potential values are assigned to the image pixels located far from the target object, and low potential values (ideally zero) to pixels located close to the object. The initialization procedure on the resulting distance map represents the second stage of MACDE, where a polar coordinate system is generated through an interactively determined seed point composed by the *x* and *y* coordinates of the pixel where it was assigned. This coordinate system divides the target object via *θ* = 2*π*/*g*, where *g* represents the degrees of each constrained polar section *S*, in which one edge sectional solution must exist. Additionally, the target object has to be confined by the spatial domain of the *n* predefined initial contours and assign *n* equidistant control points as individuals to conform one population *O*_*i*_ for each polar section *S*_*i*_. The third stage of MACDE is the segmentation process, where for each section *S*_*i*_, the DE strategy is applied to minimize the corresponding edge sectional solution by evaluating the individuals according to the external energy (fitness function) derived from (6). When the optimization process for each population is finished, the segmented object is acquired connecting the best individuals of each polar section to each other.

The procedure of the proposed MACDE image segmentation method is described as follows.

1. Compute the preprocessing step (median filter, Canny edge detector, and Euclidean distance map).
2. Initialize coordinates (*x*, *y*) of the interactive seed point, degrees *dg*, and number of snakes.
3. Initialize parameters of DE algorithm: number of generations *G*, differentiation factor *F*, and crossover rate CR.
4. Generate one population for each polar section *S*_*i*_ assigning the current snaxels as individuals.
5. For each population *O*_*i*_, we have the following.
  - For each individual *X*_*i*,*g*_, where *g* = {1,…, *G*}:
    - compute *V*_*i*,*g*+1_ by using the mutation step (9);
    - assign *U*_*i*,*g*+1_ according to the crossover operator (10);
    - apply restriction of the search space to ignore improper solutions;
    - evaluate *U*_*i*,*g*+1_ in fitness function (6);
    - update *X*_*i*,*g*+1_, if *U*_*i*,*g*+1_ is better than *X*_*i*,*g*_ by applying the selection step (11).
  - If the stopping criterion is satisfied (e.g., stability or number of generations), then stop, otherwise go to step (a).
6. Stop MACDE method.

### 3.2. Validation Metrics

To assess the medical image segmentations performed by the proposed method regarding the classical ACM and the regions outlined by two experts, Jaccard index, Dice index, and the Haussdorf distance have been adopted.

The Jaccard index *J*(*A*, *B*) and Dice index *D*(*A*, *B*) are similarity measures located in the range [0,1] used to compare binary variables [2]. These indexes are computed by using (12) and (13), respectively. In this work, the regions segmented through computational methods (MACDE and classical ACM) are represented by *A*, and *B* is used to represent the regions outlined by the experts. In these similarity measures if regions *A* and *B* are completely superimposed the obtained result is 1, and 0 when these two regions are completely different

(12) $$\begin{array}{c} J\left(A,B\right)=\frac{A\cap B}{A\cup B}, \end{array}$$

(13) $$\begin{array}{c} D\left(A,B\right)=\frac{2\left(A\cap B\right)}{A+B}. \end{array}$$

The Hausdorff distance is a widely used metric for shape matching in medical image segmentation. This metric measures the similarity between two superimposed sets by using (14), where *a* and *b* represent points defined in sets *A* and *B*, respectively, and ||*a* − *b*|| is a some underlying distance (Euclidean distance in our tests)

(14) $$\begin{array}{c} H\left(A,B\right)=\underset{a\in A}{\max}\;\underset{b\in B}{\min}||a-b||. \end{array}$$

In Section 4, the segmentation results obtained from the proposed MACDE method on different synthetic and medical images are presented and analyzed by the validation metrics.

## 4. Experimental Results

In this section, the proposed MACDE method is applied firstly, on synthetic images with several concavities and noise, and secondly, to segment the human heart and the human left ventricle from computed tomography and magnetic resonance images. The computational implementations are performed using the gcc compiler version 4.4.5 running on Debian GNU/Linux 6.0, Intel Core i3 with 2.13 Ghz and 4 Gb of memory.

### 4.1. Application on Synthetic Images

**Figure 2:**
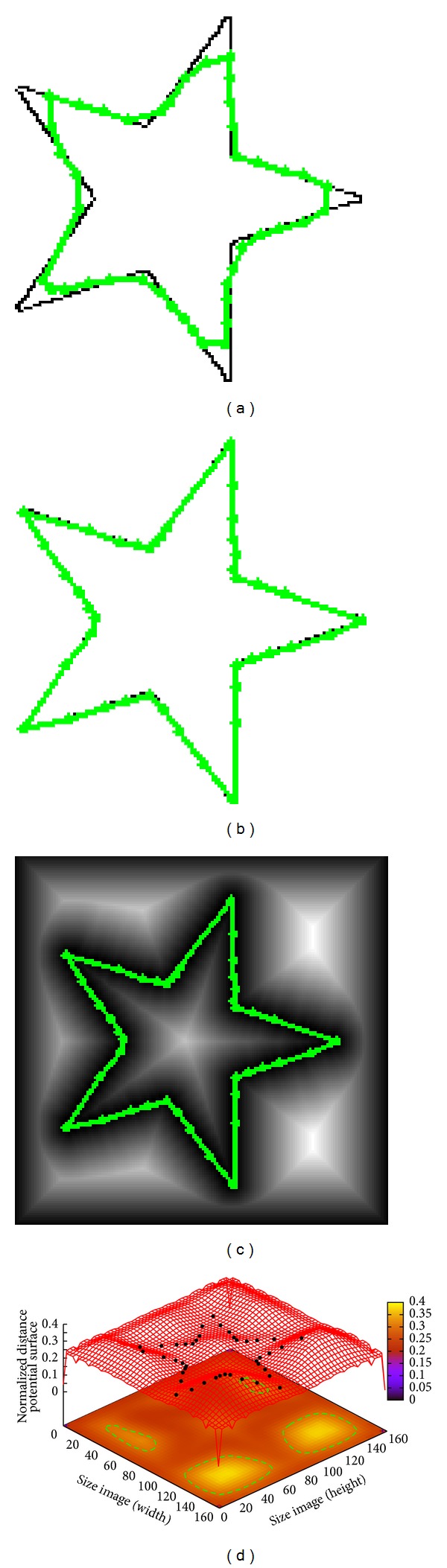
Synthetic star: (a) result of traditional ACM, (b) result of MACDE implementation, (c) result of MACDE on the Euclidean distance map, and (d) MACDE optimization process on the distance potential surface.

In Figure 2 an image of size 160 × 160 pixels containing an artificial star is presented. The segmentation result obtained by classical ACM implementation using 42 control points is shown in Figure 2(a). The ACM parameters are set as *α* = 0.01, *β* = 0.9, and *γ* = 0.05 giving an executing time of 0.090 s. In this figure the ACM implementation cannot overcome the concavity problem to fit the star boundary, which is solved through MACDE implementation as shown in Figure 2(b). In this simulation MACDE parameters are set as *G* = 10, *F* = 0.1, CR = 0.8, *dg* = 15, and number of snakes = 15, with an executing time of the optimization process of 0.140 s. The MACDE segmentation result on the Euclidean distance map is presented in Figure 2(c). This distance map is also represented as the 3D distance potential surface, in which the convergence of the optimized control points is illustrated in Figure 2(d).

**Figure 3:**
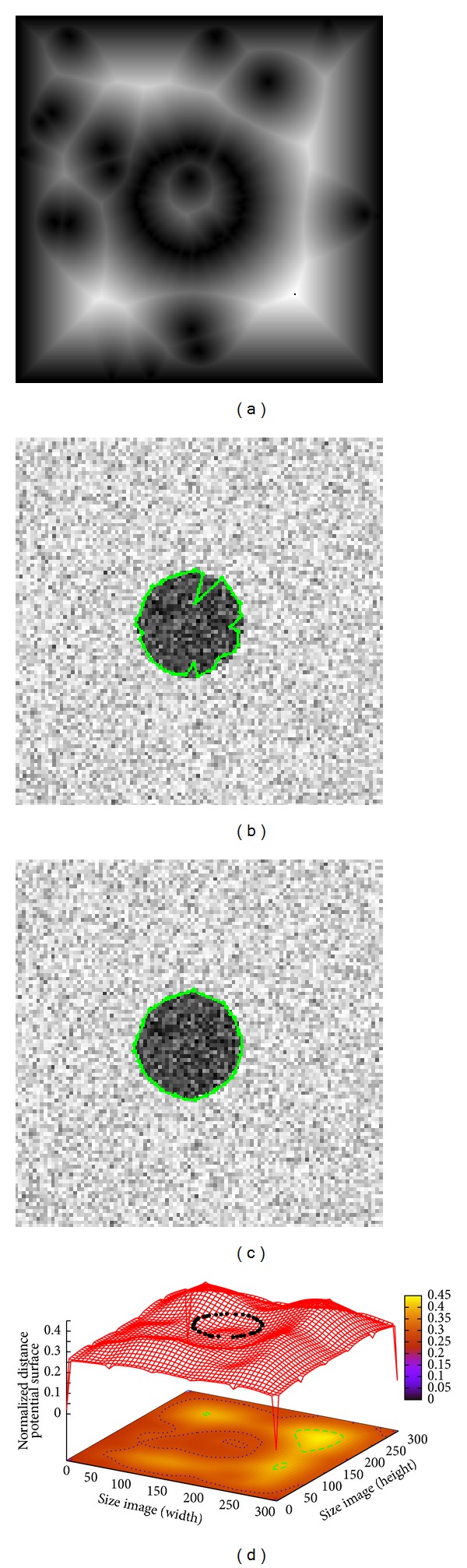
Noisy circle: (a) Euclidean distance map of the original image, (b) result of traditional ACM, (c) result of MACDE implementation, and (d) MACDE optimization process on the distance potential surface.

Figure 3 introduces an image of size 300 × 300 pixels consisting of a circle with Gaussian noise (*μ* = 0, *σ* = 0.04). The Euclidean distance map shown in Figure 3(a) is computed from the original image and illustrates the local minima problem present in the test image. The result obtained by classical ACM using 42 control points is shown in Figure 3(b), where due to the noise it cannot adjust the circle boundary accurately. The ACM parameters are set as *α* = 0.01, *β* = 0.9, and *γ* = 0.05 requiring an executing time of 0.104 s. On the other hand, MACDE method can solve the local minima problem and locate the circle boundary accurately as shown in Figure 3(c). The optimized control points on the distance potential surface are presented in Figure 3(d) which are acquired with the MACDE parameters *G* = 10, *F* = 0.1, CR = 0.8, *dg* = 15, number of snakes = 15, and the optimization process is performed with an executing time of 0.138 s.

**Figure 4:**
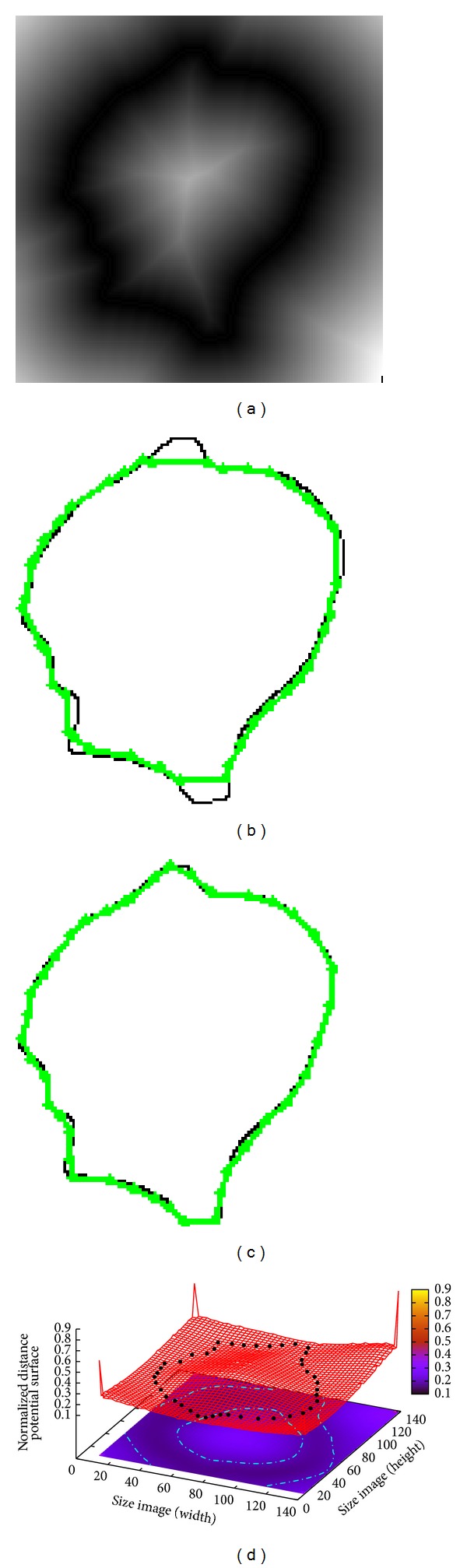
Synthetic object: (a) Euclidean distance map of the original image, (b) result of traditional ACM, (c) result of MACDE implementation, and (d) result of MACDE optimization process on the distance potential surface.

In Figure 4 an image of 150 × 150 pixels containing a synthetic object is introduced. The Euclidean distance map derived from the original image is presented in Figure 4(a) where the concavity problem is clearly evident. The segmentation result obtained by classical ACM using 42 control points cannot find the concavities of the object as shown in Figure 4(b). The ACM parameters of this simulation are set as *α* = 0.01, *β* = 0.9, and *γ* = 0.05 requiring an executing time of 0.101 s. Moreover, MACDE method can adjust the object boundary overcoming the concavity problem as illustrated in Figure 4(c) and in the distance potential surface in Figure 4(d). The MACDE parameters used in this test are set as *G* = 10, *F* = 0.1, CR = 0.8, *dg* = 15, and number of snakes = 15, achieving the optimization process in an executing time of 0.142 s.

The use of differential evolution in MACDE method provides robustness and accuracy in the three synthetic test images regarding classical ACM. Even though the computational time of the optimization process performed by MACDE is competitive with the segmentation process carried out by the traditional ACM, the proposed method improves the segmentation results avoiding local minima and concavity problems. In Section 4.2, MACDE is applied on cardiac medical images, and the segmentation results are evaluated through different distance and similarity measures.

### 4.2. Application on Medical Images

In this section, MACDE method is used in the segmentation of the human heart and the human left ventricle from datasets of sequential CT and MR images, respectively. The CT images have been supplied by the Mexican Social Security Institute, and the MR images have been provided by the Auckland MRI Research Group, University of Auckland.

**Figure 5:**
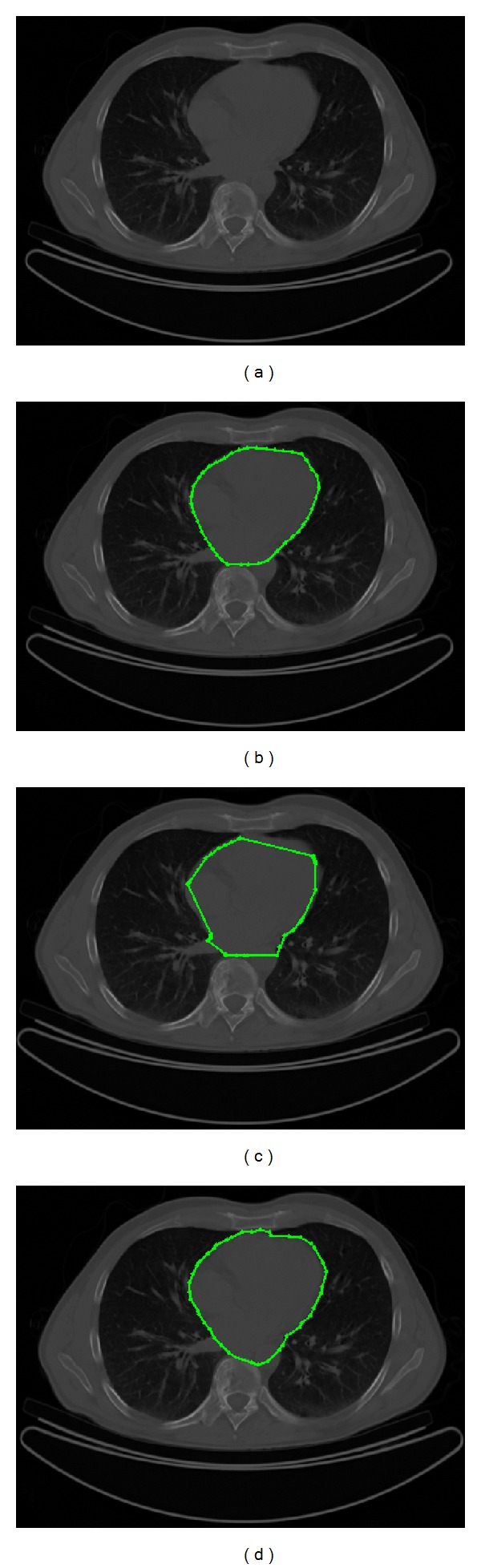
CT image: (a) test image, (b) the human heart outlined by experts, (c) result of traditional ACM, and (d) result of MACDE implementation.

In Figure 5(a) a 512 × 512 pixels CT image is presented in order to compare the human heart segmentation obtained by cardiologists in Figure 5(b), by applying the classical ACM in Figure 5(c), and by using MACDE method in Figure 5(d). The ACM parameters are set as *α* = 0.01, *β* = 0.9, and *γ* = 0.05, number of control points = 49, requiring an execution time of 0.157 s. Moreover, the MACDE segmentation fits the heart boundary appropriately in contrast to ACM, according to the manual delineation by experts, using parameters *G* = 10, *F* = 0.1, CR = 0.8, *dg* = 13, and number of snakes = 12, achieving the optimization process in an executing time of 0.212 s.

**Figure 6:**
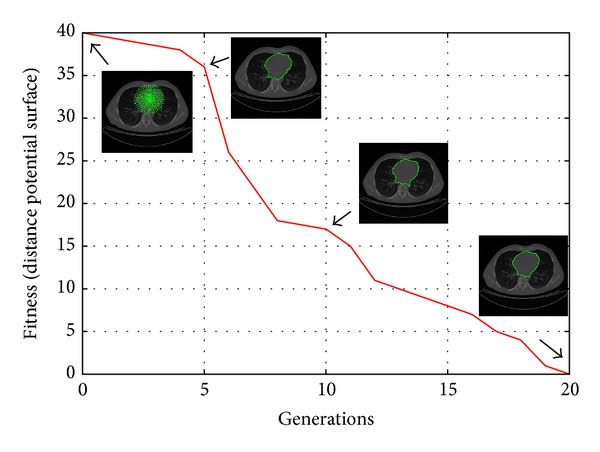
Convergence of the human heart segmentation through DE iterations.

Figure 6 shows the process of convergence of MACDE method in the human heart segmentation on a CT image. This convergence is computed through generations using the average fitness of the individuals on the distance potential surface.

**Figure 7:**
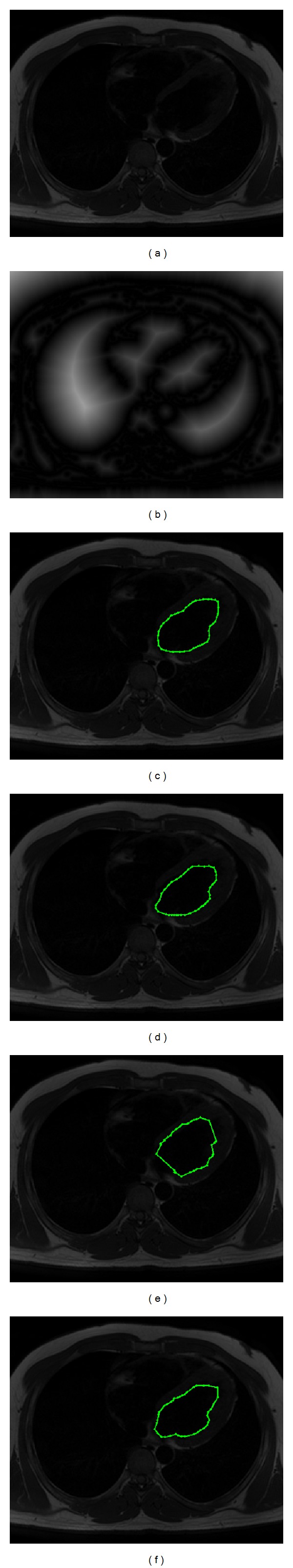
MR image: (a) test image (b) Euclidean distance map of test image, (c) the human left ventricle outlined by expert 1, (d) the human left ventricle outlined by expert 2, (e) result of traditional ACM, and (f) result of MACDE implementation.

**Figure 8:**
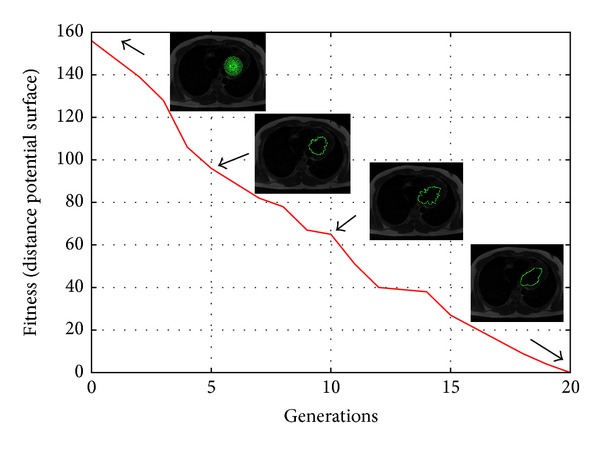
Convergence of the human left ventricle segmentation through DE iterations.

In order to introduce the human left ventricle segmentation task, in Figure 7(a) a low-contrast, 512 × 512 pixels MR image is shown.  Figure 7(b) shows the Euclidean distance map computed from the test image to get a better approximation of the search space where the computational techniques perform the optimization process. Besides, in Figures 7(c) and 7(d) the manual delineation performed by expert 1 and expert 2, respectively, are presented.  Figure 7(e) illustrates the segmentation result applying the classical ACM with parameters set as *α* = 0.01, *β* = 0.9, *γ* = 0.05, and number of control points = 45, involving an execution time of 0.235 s. Finally, Figure 7(f) presents the MACDE segmentation result, which fits the left ventricle accurately with parameters set as *G* = 10, *F* = 0.1, CR = 0.8, *dg* = 14, and number of snakes = 12. The optimization process performed by MACDE in this test image involved an executing time of 0.295 s, and it is presented in Figure 8, where the convergence is calculated through generations using the average fitness of individuals.

The initialization methodology of MACDE allows work with sequential images easily, since just the seed point coordinates (*x*, *y*) and the initial parameters are required for segmenting the whole set of images. This is an advantage over the classical ACM, because in MACDE only one user interaction is needed, while in ACM each control point is generally provided by the user resulting in a laborious task.

**Figure 9:**
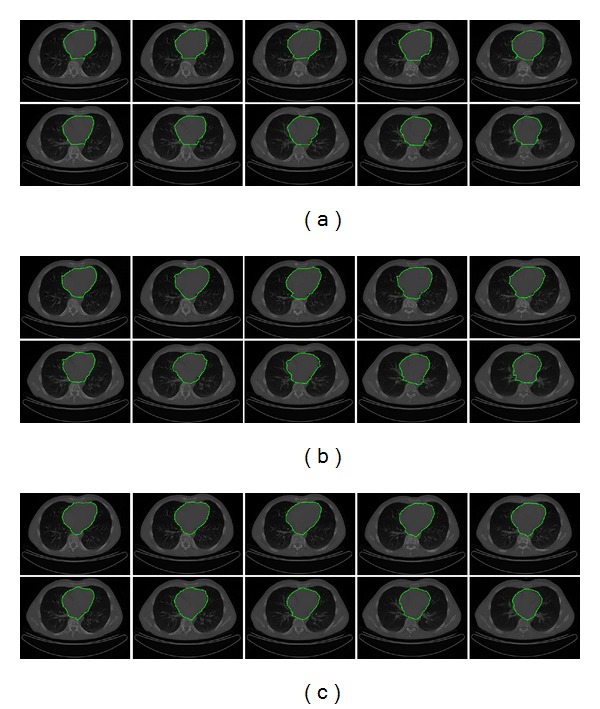
CT images (human heart segmentation): (a) results of traditional ACM, (b) results of Tseng method, and (c) results of MACDE implementation.

In Figure 9 the human heart segmentation results on a subset of CT images are presented. The whole dataset consists of 144 CT images of size 512 × 512 pixels from different patients.  Figure 9(a) illustrates the segmentation results obtained by classical ACM, where the concavity problem is clearly shown. The ACM parameters are set as *α* = 0.01, *β* = 0.9, *γ* = 0.05, and control points = 45, requiring an average executing time of 0.163 s per image.  Figure 9(b) shows the human heart segmentations obtained with the interactive Tseng method. The parameters of this simulation were tuned according to [17] as 45 control points, 9 particles for each swarm, and window size 30 × 30 pixels, given an average executing time of 0.176 s per image.  Figure 9(c) presents the segmentation results obtained by MACDE, which fit to heart boundary in a suitable way. The MACDE parameters are set as *G* = 10, *F* = 0.1, CR = 0.8, *dg* = 15, and number of snakes = 12, involving an average execution time of 0.194 s per image. On the other hand, the average similarity measures listed in Table 1 are used to assess the regions segmented by computational methods and manual delineations by experts, which indicates that MACDE segmentation method is promising in human heart segmentation.

**Figure 10:**
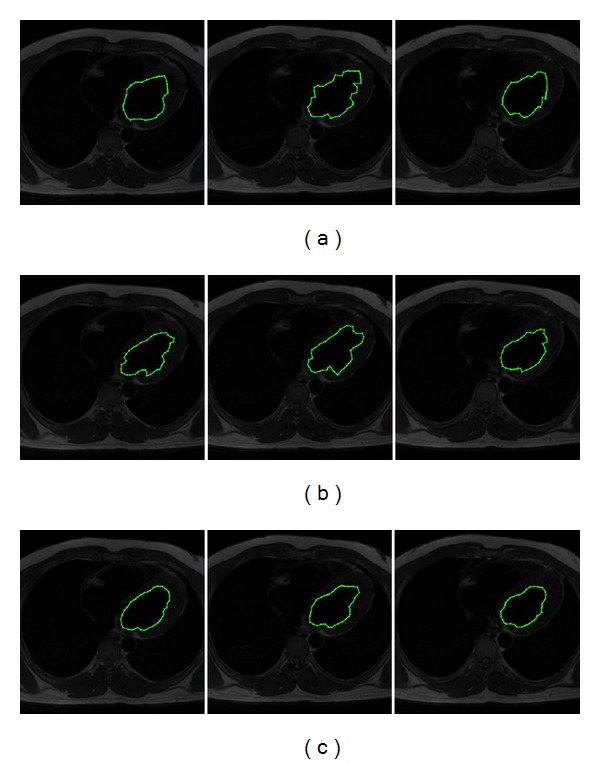
MR images (human left ventricle segmentation): (a) results of classical ACM, (b) results of Tseng method, and (c) results of MACDE implementation.

In Figure 10 the human left ventricle segmentation results on a subset of MR images are presented. The whole dataset is composed by 23 MR images of size 512 × 512 pixels.  Figure 10(a) shows the segmentation results obtained with classical ACM, where the local minima problem leads to an inaccurate convergence to left ventricle boundary. The ACM parameters in these tests are set as *α* = 0.01, *β* = 0.9, *γ* = 0.05, and control points = 42, demanding an average execution time of 0.221 s per image.  Figure 10(b) shows the segmentation results by applying the Tseng method. The parameters of this simulation were experimentally chosen as 42 control points, window size as 30 × 30, and 15 particles for each swarm, given an average executing time of 0.253 s per image. In Figure 10(c) the regions segmented by MACDE are illustrated. These segmentation results fit properly to the left ventricle boundary with parameters set as *G* = 10, *F* = 0.1, CR = 0.8, *dg* = 15, and number of snakes = 12, requiring an average execution time of 0.275 s per image. Moreover, to quantify the segmentation results, Table 2 presents the comparative analysis through Dice index, Jaccard index, and Haussdorf distance between computational methods and manual delineations by experts. This similarity analysis suggests that MACDE is competitive regarding regions outlined by experts, and it is more accurate than the classical ACM, and the interactive Tseng method.

**Figure 11:**
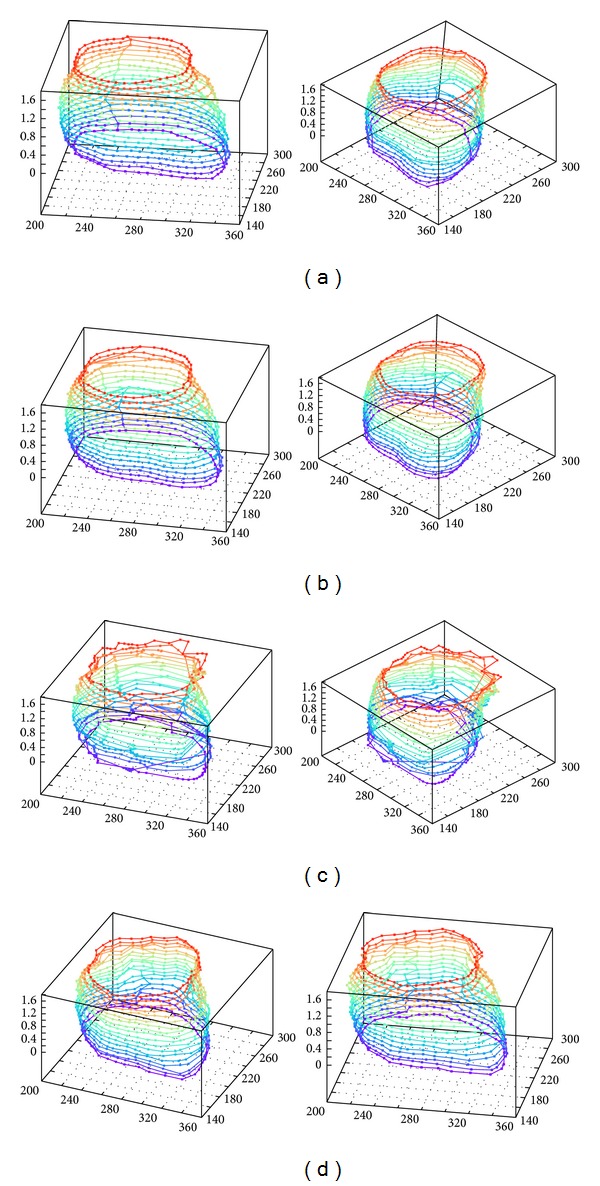
3D reconstruction of human heart from CT images: (a) result obtained from expert 1, (b) result obtained from expert 2, (c) result with classical ACM, and (d) result of MACDE method.

Finally, in order to visualize the segmentation results acquired from sequential CT images, 3D reconstruction approaches obtained from the experts, classical ACM and the proposed MACDE method are presented in Figure 11. The quality of the 3D reconstruction depends on the number of sequential images, and the approaches presented below consist of 18 CT images, which are achieved through superposition of the resulting contours according to the image acquisition order. These reconstructions illustrate a significant effectiveness and stability of MACDE in the human heart segmentation.

## 5. Conclusion

In this paper, a novel image segmentation method based on multiple active contours guided by differential evolution (MACDE) has been proposed. The segmentation method has introduced some important advantages regarding the classical active contour model and the interactive Tseng method, in particular, the partitioning of the region of interest in polar sections to overcome the local minima problem and the sensitivity to initial contour position. In order to evaluate the performance of the proposed method, some experiments with synthetic images following by experiments with cardiac medical images acquired from the computed tomography and magnetic resonance procedures were presented. The experimental results demonstrated the efficiency and stability of MACDE in the presence of noise and deep concavities. These advantages made it possible to attain a high accuracy in the human heart and human left ventricle segmentations compared to the regions outlined by experts according to the evidence showed by the set of similarity metrics. In addition, the experimental results have also revealed that MACDE is highly suitable for medical image applications, including the segmentation of sequential medical images within a competitive computational time.

## Figures and Tables

**Table 1:** Average similarity measure with Hausdorff distance, Jaccard index, and Dice index among the regions segmented by classical active contour model (ACM), interactive Tseng method, our proposed method (MACDE), and the regions outlined by two experts from the set of CT images.

Comparative studies	Distance/similarity measure		
	Hausdorff (H)	Jaccard index (J)	Dice index (D)
ACM versus Expert 1	4.0	0.3548	0.5238
ACM versus Expert 2	3.0	0.5272	0.6904
Tseng versus Expert 1	2.236	0.8260	0.9047
Tseng versus Expert 2	2.8284	0.7872	0.8809
MACDE versus Expert 1	3.0	0.8666	0.9285
MACDE versus Expert 2	1.4142	0.9090	0.9523

**Table 2:** Average similarity measure with Hausdorff distance, Jaccard index, and Dice index among the regions segmented by classical active contour (ACM), interactive Tseng method, our proposed method (MACDE), and the regions outlined by two experts from the set of MR images.

Comparative studies	Distance/similarity measure		
	Hausdorff (*H*)	Jaccard index (*J*)	Dice index (*D*)
ACM versus Expert 1	5.0	0.3548	0.5238
ACM versus Expert 2	10.4403	0.4237	0.5952
Tseng versus Expert 1	1.0	0.9090	0.9523
Tseng versus Expert 2	3.6055	0.8260	0.9047
MACDE versus Expert 1	1.0	0.8666	0.9285
MACDE versus Expert 2	3.1622	0.9534	0.9761
